# Characterization of Lipid Extracts from Different Colors of Peach Palm Fruits—Red, Yellow, Green, and White—Obtained through Ultrasound-Assisted Green Extraction

**DOI:** 10.3390/foods13101475

**Published:** 2024-05-10

**Authors:** Mayara Priscila Lima dos Santos, Orquídea Vasconcelos dos Santos, Leyvison Rafael Vieira da Conceição, Barbara Elisabeth Teixeira-Costa, Lúcia de Fátima Henriques Lourenço, Consuelo Lucia Lima de Sousa

**Affiliations:** 1 Programa de Pós-Graduação em Ciência e Tecnologia dos Alimentos, Instituto de Tecnologia, Universidade Federal do Pará, Belém 66075-110, PA, Brazilorquideavs@ufpa.br (O.V.d.S.);; 2 Programa de Pós-Graduação em Engenharia Química, Instituto de Ciências Exatas, Universidade Federal do Pará, Belém 66075-110, PA, Brazil; 3 Programa de Pós-Graduação em Biotecnologia, Universidade Federal do Amazonas, Manaus 69067-005, AM, Brazil; 4 Faculdade de Nutrição Emília de Jesus Ferreiro, Universidade Federal Fluminense, Niterói 24020-140, RJ, Brazil

**Keywords:** peach palm fruit, green extraction, fatty acids, ultrasound-assisted extraction, *Bactris gasipaes*, carotenes, edible lipid extract

## Abstract

This study represents a pioneering investigation and comparative analysis of lipid extracts from four different colors of peach palm (*Bactris gasipaes* Kunt) fruits—red, yellow, green, and white—by employing a green method based on ethanolic ultrasound-assisted extraction. This study examined the extraction yield, physico-chemical-quality attributes, chromatographic profiles (GC), color measurements, total carotenoid content, differential thermogravimetry (TG/DTA), and infrared spectroscopy (FTIR). The obtained lipid extracts displayed a high quality, considering the physico-chemical parameters of the *Codex Alimentarius*, and a fatty acids profile characterized by unsaturated fatty acids, notably omegas (ω-3, ω-6, and ω-9). The indices of atherogenicity (A.I.), thrombogenicity (I.T.), and hypocholesterolemic and hypercholesterolemic ratios revealed superior outcomes for the red peach palm lipid extract (approximately 0.35, 0.52, and 2.75, respectively), along with higher levels of β-carotene (748.36 µg of β-carotene per 100 g^−1^ of lipid extract) compared to the yellow, green, and white counterparts. Consequently, this research successfully demonstrates the efficacy of using a green extraction method in preserving the lipid’s quality, which can display cardiovascular functionality and thermal stability. These findings underscore the considerable potential of peach palm lipid extract as a valuable raw material for diverse industrial applications across various sectors. The results support its utilization in the production of functional food products and nutraceuticals due to its favorable fatty acid composition, potent antioxidant properties exhibited by its high β-carotene content, and notable cardiovascular functionality indices.

## 1. Introduction

Amazonian fruits and seeds, rich in edible vegetable oils and fats, have been studied over the years, given their nutritional composition; bioactive compounds, i.e., polyphenols; vitamins, and other substances with health-related properties [1,2,3,4]. The Amazonian rainforest region hosts a significant fruit species that have potential to be exploited as a source of bioactive and functional foods, among which, the peach palm fruit (*Bactris gasipaes*) stands out. Vegetable oils from fruits and nuts have been used by human beings, as well as by the food, cosmetic, and pharmaceutical industries, since ancient times, to develop different functional and nutritional products [5]. The use and consumption of peach palm fruits in regional culinary dishes are very popular in Northern Brazil and they are commonly found in local trade street markets. *Bactris gasipaes* Kunth is a palm tree that belongs to the Arecaceae family [6]. It was reported to be domesticated by indigenous people in pre-Columbian times in southwestern Amazonia, primarily used for logging purposes, and only later used for the extraction of oil fruit [7]. Approximately 73 species are found in the Americas, especially in South Mexico, the Caribbean region, Brazil, Bolivia, and Paraguay [8].

Peach palm fruits are drupes with 3–4 cm diameters, one seed, a thin epicarp with colors varying from white to orange–red, and a mesocarp rich in vitamin A, which explains its yellowish, orange, or reddish color [2,6,7,9]. The peach palm tree can produce up to 10 bunches per year/plant, which can have around 12 kg of fruits [10]. The production of peach palm fruits in Brazil reached 8873 tons in 2017 [11]. Despite the relatively moderate production of the fruit, the peach palm tree has a greater production result related to the palm heart, commonly known as *palmito de pupunha*, which has a production value close to 109,200 tons [12]. The peach palm fruit is commonly consumed after cooking in boiling water, eating its mesocarp as a source of fiber and vitamins, or using it for other culinary purposes [7]. Peach palm fruit can be considered an oily fruit due to its high content of lipids, which can reach 8–41%, with a significant content of unsaturated fatty acids (up to 36%) [8,13]. Other important nutrients found in the pulp of peach palm fruits are carbohydrates (24–44%), proteins (3.9–5%), dietary fiber (4.8–10%), and minerals (2.7–3.9%) [13]. The main fatty acids in peach palm pulp oil are lauric acid (33–60%) followed by myristic acid (18.9–27.8%), oleic acid (21.9–24.3%), and palmitic acid (6–9.6%) [3]. The oil from the pulp of peach palm fruits displays different contents of bioactive substances, such as total tocopherols (59 mg kg^−1^ oil), total carotenes (357.42 mg kg^−1^ oil), phytosterols (4456 mg kg^−1^ oil), and β-sitosterol (51–55.0 mg kg^−1^ oil) [3]. This highlights the potential of the peach palm fruit as a source of lipids and carbohydrates to diversify food applications.

It is known that the content of lipids and their composition of fatty acids in vegetable sources are influenced by several factors, mainly related to their botanical origin, genetic variations, climate and soil characteristics, planting management, processing, and extraction conditions, and refinement steps [3,14,15]. Traditionally, lipids’ extraction by using conventional methods is well-stablished and easy to perform; however, it has some important drawbacks, such as high time-consumption, the use of elevated temperatures, toxicity from solvents, and need for recovery and purification steps, which can significantly influence the nutritional quality and stability of oils and their derived products [5]. In this context, minimizing the negative effects on lipid extraction using new methods is an objective of green chemistry [5]. Supercritical and subcritical fluid extractions and microwave- and ultrasound-assisted extractions are innovative techniques to produce vegetable oils with higher nutritional, sensorial, and sustainable qualities [5,16,17]. Thus, the extraction of natural products, such as vegetable oils and their bioactive compounds, using green methods meets two of the major challenges of the 21st century, protection of life (both consumers and environment), and meanwhile enhances industry competition for novel markets [18].

Ultrasound technology is among the green technologies that can be used for homogenization, emulsification, extraction, and other activate transfer phenomena [18,19]. This technology has been known since the 1880s; however, it was commonly applied to the sonochemistry field [18,19]. When used for the extraction of natural compounds, such as polyphenols and vegetables oils, the experimental conditions can be diverse. Therefore, a common finding among the researchers is that ultrasound-assisted extraction (UAE) is more time-effective when extracting biological substances than traditional methods [19]. Ultrasound consists of sonic waves that can deform solid, liquid, or gaseous media by the cavitation phenomenon, which generates cavitation bubbles [19]. Ultrasound can operate in a frequency range from 20 kHz to 10 MHz, which allows us to differentiate two types of ultrasounds—diagnostic and power [18,19]. The formation of cavitation bubbles grows and decreases during rarefaction and compression cycles, respectively, until it reaches a critical diameter that collapses and releases energy [19]. The collapse of cavitation bubbles on the surface of a solid material, such as a cell wall matrix, increases local pressure and temperature, which destroy the cell wall and release its internal content into the extraction media [19]. Thus, UAE has improved the quality of extracted material, reduced the energy consumption, extracted bioactive compounds in aqueous media, and obtained substances with more efficiency. UAE is considered an advantageous technique and an environmentally friendly method, specifically because of the equipment energy efficiency, potential use of less toxic solvents, and the employment of mild extraction temperatures, which have fewer negative impacts on the sample properties, such as oxidation and stability status [18,19]. UAE and microwave-assisted extraction were recently used for the isolation of polyphenols and bioactive substances from açaí berries [20]. The use of UAE to obtain lipid extract from peach palm pulp is a novel approach. Recently, some researchers used UAE to efficiently extract up to 67 mg of carotenoids per 100 g of peel from peach palm fruits [21]. This highlights the potential use of UAE for such unconventional fruits, such as peach palm. Furthermore, the use of green extraction techniques complies with the global guidelines for reducing the use of hazardous solvents in the industry by the Food and Agriculture Organization (FAO) and environmental legislation of the European Union [22].

In this context, this work aimed to obtain lipid extracts from the pulp of four differently colored peach palm fruits (*Bactris gasipaes*)—red, yellow, green, and white—using ethanolic-based ultrasound-assisted extraction. The extraction yield, physico-chemical-quality characteristics, chromatographic profiles (CGs), and total carotenoid content of the extracts were evaluated and colorimetric, differential thermogravimetric (TG/DTA), and FTIR spectral analyses were also performed.

## 2. Materials and Methods

### 2.1. Materials

Four differently colored peach palm fruits (*Bactris gasipaes*)—red, yellow, green, and white—were acquired from a free market located in the metropolitan region of Belém, State of Pará, Brazil (latitude: −1.45502, longitude: −48.5024, 1°27′18′′ South, 48°30′9′′ West). For each color, five bunches (close to 4 kg of fruits), totaling approximately 20 kg of fruits were purchased, which were harvested from September to December 2023 (according to their producer). The bunches were transported in low-density polyethylene plastic bags to the Food Sciences Laboratory, Faculty of Nutrition, Federal University of Pará, where the fruits were visually selected to eliminate contaminants, and then sanitized (sodium hypochlorite solution at 100 μg mL^−1^ for 15 min). The fruits were left to dry naturally at room temperature and protected from light and humidity. The whole fruits of each color were manually classified using the quartering technique to obtain representative laboratory samples (±one kilogram for each color of fruit). All the chemicals used in this study were of analytical grade purchased from Sigma-Aldrich Brazil Ltd. (São Paulo, SP, Brazil). This research was registered with access activity in the National System for Management of Genetic Heritage and Associated Traditional Knowledge (SisGen, AA5BEE7). To ensure the proper taxonomic identification of the plant, the register of the plant (*Bactris gasipaes*), n° 4070, in the HF Professora Normélia Vasconcelos Herbarium was accessed.

### 2.2. Sample Preparation

The fruit samples of each color were separately cooked in boiled water using a domestic pressure pan for 15 min and left to naturally cool down at room temperature. After this, the fruits were manually peeled with a stainless-steel knife. The cooked peach palm pulps were subjected to freeze-drying (Solab, model SL-404B, Piracicaba, SP, Brazil) until the samples displayed a moisture content of around 6% ± 1 (w.b.); then, they were crushed in a Willye knife mill (Fortinox brand, model Start FT 50, Guarulhos, SP, Brazil) and granulometric separation was performed in a digital electromagnetic sieve shaker (Bertel, model VP-01, São Paulo, Brazil). The particle size of the ground samples was standardized using a sieve of 250 µm. Subsequently, the samples were vacuum packed (Cetro, model DZ-280, Rio de Janeiro, RJ, Brazil) and stored in a refrigeration environment until analysis.

### 2.3. Extraction and Yield of Peach Palm Lipid Extracts

Ultrasound-assisted extraction was performed according to Ordóñez-Santos et al. [22], and preliminary tests were conducted, following the best parameters obtained in terms of yield in the ultrasound-assisted extraction of peach palm lipid extracts. Ethanol 99.9% (M = 46.07 g/mol) was used as the green solvent for the extraction procedures. The ground fruit pulps were weighed (5 g) using an analytical balance and placed in an amber Erlenmeyer flask; then, the extraction solvent was added at a 1:5 (sample weight:solvent volume) ratio. The sample:solvent mixture was subjected to the extraction procedure in an ultrasound bath with an integrated temperature-controlled system (Solid Steel, model SSBuc 15 L, Piracicaba, SP, Brazil) for 30 min at a frequency of 20 kHz and a temperature of 50 ± 2 °C. After that, the ethanolic extracts were centrifuged at 3600 rpm to separate the insoluble fraction/micelles. The supernatant fraction of the extracts was vacuum filtered in quantitative filter paper and left to dry in a desiccant container at 50 °C. After solvent evaporation, the resulting material was considered and named as a lipid extract from four different peach palm fruits: red (RLE), yellow (YLE), green (GLE), and white (WLE). The oil samples were placed in an amber container at a temperature of 7 °C and stored until further analysis [23].

The lipid extract yield was calculated according to Equation (1):

(1) $$\%Yield=\left(\frac{W_{extract}}{W_{pulp}}\right)\times 100$$

where $W_{extract}$ is the weight of the lipid extract (in grams) and $W_{pulp}$ is the weight of the freeze-dried peach palm pulp (in grams).

### 2.4. Fatty Acid Profile Analysis

The profile of fatty acids in lipid extracts was established by the methyl esterification of fatty acids in accordance with the methodological recommendation of the International Organization for Standardization ISO 5509 [24]. After phase separation, the collected supernatant was used for gas chromatography analysis (GC Varian 430, California, USA) equipped with a microcomputer using Galaxie Chromatography software (Data System Version 1.9.3.2 software, Varian Inc., Palo Alto, California, USA) based on the following chromatographic conditions: fused silica capillary column SP^®^-2560 (Supelco, Bellefonte, PA, USA) (100 m long × 0.25 mm internal diameter) containing 0.2 μm of polyethylene glycol. The operating conditions were: fractional injection, 50:1 ratio; column temperature at 140 °C for 5 min, programmed with an increasing rate of 4 °C per min up to 240 °C, carrier gas: helium, isobaric pressure of 37 psi, linear velocity of 20 cm/s; compensation gas: helium at 29 mL/minute; injector temperature of 250 °C (model: Varian CP 8410) (auto-sampler); and detector temperature of 250 °C. Internal standards, C15:0—methyl pentadecanote, and 37-Component FAME Mix (methyl esters of fatty acids ranging from C4 to C24 CRM47885—from Sigma–Aldrich, Milan, Italy) were used. Qualitative composition was determined by comparing the peak retention time with respective fatty acid profiles. The quantitative composition was carried out by area normalization expressed as a percentage of mass as established by the official method (Ce 1-62) [25].

### 2.5. Functional Quality of Lipid Extracts

The functionality of lipid extracts was established based on their respective fatty acids profiles, by using three indices: (1) atherogenicity index (AI), (2) thrombogenicity index (TI) according to Ulbricht and Southgate [26], and (3) the hypocholesterolemic index hypercholesterolemic ratio (H/H) proposed by Chen et al. [27]. The FA content in peach palm lipid extract was classified into fractions according to the presence and number of double or triple bonds, as saturated fatty acids (SFAs), unsaturated fatty acids (UFAs), monounsaturated fatty acids (MUFAs), and polyunsaturated fatty acids (PUFAs). The following equations were used to calculate these indices:

(2) $$AI=\frac{\left[(C_{12:0}+\left(4\times C_{14:0}\right)+C_{16:0})\right]}{\left(PUFA+MUFA\right)}$$

(3) $$TI=\frac{\left(C_{12:0}+C_{16:0}+C_{18:0}\right)}{\left[(0.5\times MUFA)+(0.5\times n6PUFA)+(3\times n3PUFA)+(\frac{n3PUFA}{n6PUFA})\right]}$$

(4) $$H/H=\frac{C_{18:1}+PUFA}{\left({C_{12:0}+C}_{14:0}+C_{16:0}\right)}$$

### 2.6. Quality Parameters

The physicochemical quality of peach palm lipid extract was evaluated for its agreement with the standard legislation for oils and fats, such as the American Oil Chemists’ Society (AOCS) standard methods. Acidity and peroxide indices of the peach palm extracts, as sources of edible lipids, were determined using the Cd 3d-63 and Cd 8-53 methods, respectively [25]. For the determination of the acid value, 2 mL of a 1% phenolphthalein indicator, 125 mL of a solvent mixture (equal parts of isopropyl alcohol and toluene), and 10 g of sample were added to an Erlenmeyer flask and homogenized until complete sample dissolution. This mixture was titrated with a 0.1 M potassium hydroxide solution. Blank samples were prepared without the lipid extract. The acid value was determined by the following Equation (5):

(5) $$Acid\;Value,\;mg\;KOH/g\;of\;sample=\frac{\left(A-B\right)\times M\times 56.1}{W}$$

where A = mL (volume) of standard alkali solution in titration.
B = mL (volume) of standard alkali solution in titration of the blank.
M = molarity of the standard alkali solution.
W = g of the sample (mass).

For the determination of the peroxide value, approximately 5 g of sample and 30 mL of a 3:2 acetic acid:chloroform solution was added to an Erlenmeyer flask and homogenized; then, 0.5 mL of a saturated iodine solution was added. The mixture was homogenized, and 30 mL of distilled water was added, followed by titration with 0.01 N of a sodium thiosulfate solution, using 2 mL of a 1% starch solution as the indicator. The peroxide value was determined by the following Equation (6):

(6) $$PeroxideValue,mgKOH/gofsample=\frac{\left(S-B\right)\times N\times 1000}{W}$$

where S = mL (volume) of standard alkali solution in titration.
B = mL (volume) of standard alkali solution in titration of the blank.
N = normality of the sodium thiosulfate solution.
W = g of the sample (mass).

### 2.7. Color Measurements

The color measurements were carried out as a comparative parameter between the lipid extracts by a tristimulus colorimeter (Konica Minolta, model CR-400, Japan), using a CIELAB system with the following operating conditions: diffuse lighting/0° viewing angle and D65 lighting source [28]. The following parameters were obtained: L*, a*, and b*, where L* is related to luminosity (black = 0 and white = 100) and indices a* and b* correspond to chromaticity (+a = red and −a = green; +b = yellow and −b = blue). The colors of the samples were compared using the whiteness index (WI), which indicates how close a sample is to an ideal white ((WI = 100) (Equation (6)). The h_ab_ index indicates the chromaticity (0°, red; 90°, yellow; 180°, green; 270°, blue) (Equation (8)). These calculations followed those proposed by Palavecino et al. [29].

(7) $$WI=100-\sqrt{{\left(100-L^{*}\right)}^{2}+{\left(a^{*}\right)}^{2}+{\left(b^{*}\right)}^{2}}$$

(8) $$C^{*}=\sqrt{{\left(a^{*}\right)}^{2}+{\left(b^{*}\right)}^{2}}$$

(9) $$h={tan}^{-1}b^{*}/a^{*}$$

(10) $$∆E=\sqrt{{\left(L_{1}-L_{2}\right)}^{2}+{\left(a_{1}-a_{2}\right)}^{2}+{\left(b_{1}-b_{2}\right)}^{2}}$$

### 2.8. Determination of Total Carotenoids

Total carotenoid levels were quantified according to the method carried out by Rodriguez-Amaya [30], with adaptations. An aliquot of peach palm lipid extract was mixed with 10 mL of petroleum ether; then, the absorbance of the resulting mixture was quantified in a spectrophotometer at 450 nm. The carotenoid content, expressed as β-carotene, was calculated by the following equations:

(11) $$X\;(\mu g)=\frac{A\times V\times 10^{6}}{A_{PE}{\;\times \;m}_{0}\times 100}$$

(12) $$\beta \text{-}\mathrm{caroten}e\;(\mu g/g)=\frac{X\;(\mu g)}{S(g)}$$

where X (μg) is the weight of carotenoids, A is the absorbance at 450 nm, V is the volume (mL) of the solution that presents an absorbance of A, $m_{0}$ is equal to the mass of the sample (g), $A_{PE}$ is the absorbance of β-carotene in petroleum ether, and S is the weight of the sample (g).

### 2.9. Thermogravimetric Analysis

Thermogravimetric analysis (TGA) was used to investigate the thermal stability of the peach palm lipid extracts, and derivative (DTG) curves were used to measure and compare the peak temperatures. The analysis was carried out on a thermobalance (Shimadzu, model DTG-60, Kyoto, Japan), under the following conditions: air and nitrogen flow of 60 mL min^−1^; heating ramp of 10 °C/minute, in the temperature range of 20 to 600 °C; and alumina crucible and mass of 5 mg ± 0.5. Experimental data were analyzed with Origin 8.0 (OriginLab Corp., Northamptom, MA, USA).

### 2.10. Infrared Spectroscopy

Fourier Transform Infrared Spectroscopy (FTIR) analyses were performed on peach palm lipid extract in a Perkin Elmer spectrometer, model: Frontier 98737 (Waltham, MA, USA) at 25 °C in a wavenumber range of 4000–400 cm^−1^. Spectra were recorded by averaging 20 scans with a 4 cm^−1^ resolution in the transmission mode. Experimental data were analyzed with Origin 8.0 (OriginLab Corp., Northamptom, MA, USA).

### 2.11. Statistical Analysis

All the analyses were performed in triplicate and data expressed as mean ± standard deviation. Data were analyzed and compared using the analysis of variance (ANOVA) at a significance level of 95%; significant differences between means were established at *p* ≤ 0.05 and with the Tukey’s post hoc test. Statistical analyses were conducted using the software package Statistica^®^ Software version 8.0.

## 3. Results and Discussion

### 3.1. Extraction and Yield of Peach Palm Lipid Extracts

The lipid extract yields of the differently colored peach palm fruits—red, yellow, green, and white—are shown in Table 1.

The average yield of the ultrasound-assisted extraction of peach palm lipid extracts is in the range of 2.15–8.85%, as shown. Lipid extracts from red and yellow peach palm fruits presented the highest percentages, 8.85 ± 0.95% and 7.35 ± 0.83%, respectively. Meanwhile, lipid extracts from the green and white varieties showed lower values, 3.35 ± 0.75% and 2.15 ± 0.17%, respectively. These results show the diversity in terms of energy macronutrients in these matrices, components of the *Bactris gasipaes* botanical family. It was noted that extractions assisted by ultrasound and green solvent presented a lower yield when compared to other conventional extraction methods, such as those that use solvents derived from waste from the petrochemical industry. Studies performing solid–liquid extractions using solvents such as petroleum ether or hexane from peach palm fruits by Santos et al. [1], Torres-Vargas et al. [31], Monteiro et al. [32], and Santos et al. [33] (2022) found a higher percentage of lipid extract yield than this study. The choice of the extraction method to be applied must follow parameters that consider factors such as raw material, process efficiency, complexity or simplicity of the extraction method, cost, extraction speed, possibilities of loss or degradation, absence of chemical product waste, quality of the extracted product, and, with regard to this research, its suitability for the principles of green chemistry. Among these from an environmental perspective, ultrasound-assisted extraction with green solvents is considered a fast method, with the application of environmentally clean reagents, with little or no generation of petroleum-derived pollutant residues, presenting low toxicity [18,34]. As for the economic aspects, its value is minimized by the cost–benefit ratio of acquiring equipment and solvents derived from organic vegetable raw materials in favor of an efficient and environmentally clean method.

### 3.2. Fatty Acid Profile Analysis

The fatty acid profiles of peach palm lipid extract are presented in Table 2. The most prominent saturated fatty acid was palmitic acid, with a significant difference between the peach palm varieties, with values varying from approximately 23.8% in the red to around 42.6% in the white peach palm fruit. This result may be related to differences in the macronutrient constitution, as the white variety has a lower lipid content, probably strongly related to other macronutrients, such as sugars (glycolipids), proteins (glycoproteins), and fibers (fibroelastic), providing a lot of resistance to their extraction. Under the conditions studied, and at room temperature, a material with a more solid consistency than the lipid extracts of other varieties was obtained. Among the monounsaturated fatty acids (MUFAs), oleic fatty acid presented the highest proportion among all fatty acids, with values varying from 40.7% in the white variety to 60.2% in the red variety, presenting a different percentage among all the samples (*p* ≤ 0.05). Da Costa et al. [8] revised other studies related to the fatty acid profile in peach palm oil and found that the MUFA content ranged from 42.09 to 68.2%, majorly related to the amount of oleic acids (37.0–60.8%). As for palmitoleic fatty acid, the red and yellow varieties were significantly different, while the green and white varieties did not show different percentages (*p* ≤ 0.05). The results of the fatty acid profile show that, among the polyunsaturated class, linoleic acid, also known as omega 6 (ω-6), and linolenic acid, known as omega 3 (ω-3), deserve to be highlighted, both with values showing significant differences (*p* ≤ 0.05) between all varieties. The content of polyunsaturated fatty acids (PUFAs) in peach palm oil can range from 2.5 to 10.1%, according to Da Costa et al. [8]. The PUFA content found in the present work, 2.5–10.5%, agrees with the literature. The omega-6 value of the red peach palm, yellow peach palm and white peach palm varieties was higher than in the research by Serra et al. [35] using other Amazonian fruits, such as buriti oil—*Mauritia flexuosa*—(1.13%), patawa oil—*Oenocarpus bataua—*(3.49%), and babassu oil—*Orbignya phalerata*—(2.6%).

Higher contents of oleic and linoleic acids in vegetable oils are an indication of their good potential in terms of nutrition and health, especially because diets that replace SFAs for UFAs can be associated with lower cholesterol and, therefore, reducing the chance of having cardiovascular diseases [36]. The nutritional functionality of the constituents of the omega family (ω-6 and ω-3) is reported by the essential need for these constituents by the human body since they cannot be synthesized and must be acquired from the usual diet [37]. These substances make up a special class of molecules that are necessary for human defense metabolism as they are related to the synthesis and conversion of the basic constituents of the immune system, such as arachidonic acids, which are precursors of eicosanoids (modulators of inflammatory responses) [37]. Linolenic acid (ω-3) is a precursor of eicosapentaenoic, docosahexaenoic, and n-3 eicosanoids, compounds based on pharmacological inputs, which include prostaglandins, leukotrienes, thromboxanes, and lipoxins, related to anti-inflammatory actions and processes. In inflammatory diseases, these compounds have been applied to combat the prevention of coronary diseases, arrhythmia, and thrombosis [37,38].

### 3.3. Functional Quality of Lipid Fractions and the Content of Total Carotenoids

The hypocholesterolemic/hypercholesterolemic ratio, atherogenicity, thrombogenicity indices, and the total carotenoids content in UAE peach palm lipid extracts are listed in Table 3. These estimated nutri-functional-quality indices show the potential for the possible positive effects of their inclusion in common diets related to anti-thrombogenic, anti-atherogenic, and anti-hypercholesterolemic effects, forming good indices that provide cardio-protective effects in the human body.

The lipid extracts from the red peach palm variety presented values for the atherogenicity and thrombogenicity indices of around 0.35 and 0.52, while the white peach palm presented values of 0.78 and 1.34, respectively. This behavior for the white peach palm variety may be related to higher proportions of saturated acids, such as palmitic acid (C:16). The nutritional functionality expressed by the AI and TI can be related to anti-platelet aggregation actions, aiming to prevent, among other cardiovascular pathologies, the development of atherosclerosis and thrombosis. Pinto et al. [39] inferred that the AI and TI values should be as low as possible (close to zero), while the H/H value should be high in oils for human intake. In the present research, the lipid extract with the greatest potential for cardioprotective function was the red peach palm lipid extract, and the lipid extract with the lowest potential was the white peach palm lipid extract.

The hypocholesterolemic and hypercholesterolemic ratio index is perceived as a parameter for the prevention of cardiovascular health problems aimed directly at the relationship between cholesterol levels, helping to protect the cardiac system, and being an adjunct in the treatment of coronary diseases. Therefore, the higher this index, the better its contribution to health [37]. In this research, the highest estimated values were 2.75 for the red peach palm lipid extract, followed by the yellow variety, 1.90. These results are directly related to the fatty acid profiles presented for each peach palm variety. When comparing the H/H data of peach palm lipid extracts in this work with other studies by Santos et al. [1,33] to red, yellow, and white peach palm lipid extracts (1.16, 0.84, and 0.87, respectively) obtained using conventional solid–liquid extraction methods (Soxhlet apparatus and petroleum ether as a solvent), it is possible to observe that UAE lipid extracts display higher values, which can be an indication that this extraction method has a greater impact on the quality. Thus, it can be inferred that peach palm lipid extracts obtained by ultrasound-assisted extraction and using a green solvent have a better nutri-functional quality in terms of their fatty acid profiles and protective effects against cardiovascular diseases, when compared to lipids obtained by conventional extraction techniques.

Total carotenoid content is strongly influenced by variety, state of maturity, fruit color, and soil richness, among others. Red fruit generally stands out as a nutritious food in terms of its higher content of total carotenoids compared to fruits of other colors, which makes it possible to infer that the more intense the red–orange color, the higher the levels of carotenoids present [21,40,41]. In the present work, the content of total carotenoids varied from 748 to 32 μg of β-caroten 100 g^−1^ lipid extract. According to the Brazilian Health Legislation, RDC n° 269 of 22^nd^ September, 2005 [42], the content of total carotenoids in fruits must be converted into provitamin A, which considers that 1 μg of beta-carotene is equal to 0.167 μg of retinol equivalent (RE). In another study by Santos et al. [1], using the red peach palm variety, the total carotenoids content in its oil was 139.01 μg of RE; this datum correlates to approximately 37% of the recommended daily intake of vitamin A. Britton and Khachik [43] proposed a classification of foods based on their content of carotenoids as follows: low level (0–100 µg 100 g^−1^), moderate level (100–500 µg 100 g^−1^), high level (500–2000 µg 100 g^−1^), and very high level (≥2000 µg 100 g^−1^). Therefore, the peach palm lipid extract from the red variety can be considered as a high source of carotenoids.

### 3.4. Lipid Extract-Quality Parameter

The results for the physicochemical parameters, acidity, and peroxide indices of the lipid extracts from four colors of peach palm fruit are presented in Table 4.

Low acidity and peroxide values are usually related to the quality of edible fats and oils for human consumption [44,45]. The Codex Alimentarius [46] recommends maximum values of 4.0 mg KOH g^−1^ and 15 mEq active oxygen kg^−1^ for acidity and peroxides, respectively, in virgin fats and oils. Higher values of acidity can be an indication of hydrolytic degradation linked to a higher content of free fatty acids and, consequently, changes in oil quality [47]. Similarly, the higher values for the peroxide index in vegetables oils can be an indication of the highest content of hydroperoxides and other secondary oxidation products, which influences the acceptability of fats and oils due to their unpleasant flavor [48]. It was noted that all samples displayed low levels of acidity and peroxides, which was a good indication of their conservation status. When comparing the samples, it was observed that only the white variety showed a significant difference (*p* ≤ 0.05). This behavior may be related to the lower level of total carotenoids in the white variety of peach palm, since these compounds can act as antioxidant substances, protecting against degradation processes as well as factors inherent to the extraction process.

In the work of Santos et al. [1,33], peach palm lipid extracts obtained by using conventional extraction methods, such as solid–liquid extraction with organic solvents (petroleum ether and hexane), displayed higher levels of acidity and peroxides (1.03−2.45 mg KOH g^−1^ and 2.17−5.47 mg Eqk g^−1^, respectively). This behavior can be associated with a slightly negative effect on the quality of lipids obtained by using conventional methods, since it is known that conventional extractions use a higher temperature and longer sample exposure to other oxidative factors, such as light and humidity [47,48]. Although ultrasound-assisted extraction produced a lower yield, the lipids obtained presented a better quality related to oxidative factors measured by acidity and peroxide indices; thus, it presented less signs of oxidative degradation and better physical-chemical stability.

### 3.5. Color Measurements

Another aspect visually observed and instrumentally evaluated was the color of vegetable oils. Visually, the color of the oils is related to the consumer’s sensory perception of the food and is a decisive factor in the purchase and consumption of food products. Instrumentally, color is measured using colorimetric techniques. The instrumental colorimetric results for the lipid extracts from four colors of peach palm fruits, using the CIELAB system, are presented in Table 5.

The analysis of the color parameters evaluated for the peach palm lipid extracts shows that the a* parameter changed from 11.4 for the red sample to 2.3 for the white one. For the transition parameters (b*) the red sample showed a value of 9.7, while the yellow extract displayed 11.43, the green extract showed 10.16 and the white sample displayed a value of 4.2. The changes in these parameters are related to the peculiarities of the color of peach palm varieties researched in this study. The differences in terms of the color of the lipids represent the characteristics found in the endocarp and mesocarp parts of the fruits that were used for extraction.

The color variation shows a difference between the white peach palm lipid extract, with a lower correlation between the sample and the instrumental standard (ΔE), unlike the lipid extracts from other varieties that show similar color variations, with very close numerical averages (Table 5). The chromaticity index varies from blue (−60) to yellow (+60), which showed positive values in all samples. However, the red, yellow, and green varieties showed similar positive results (14.97; 13.05; and 11.50, respectively) with a tendency toward yellow. The white peach palm lipid extract followed the same trend, however with a much lower value (4.79). The hues of the lipid extracts were expressed by the defined hue angle (h_ab_) (0°, red; 90°, yellow; 180°, green; 270°, blue). This shows the similarity of the yellow, green, and white peach palm lipid extracts with average values of 60°, differing numerically from the red peach palm lipid extract with a tone value around 40°. Based on the points above, it can be seen that all the lipid extracts in this research have a color close to yellow.

The whiteness index for the white peach palm lipid extract stands out, with a higher result than the other varieties, with an index of 38.61 compared to the red, yellow, and green varieties; as expected, with a greater tendency toward white, the yellow and green varieties have similar values, and the red variety presents the smallest difference with a lower average whiteness value of 25.57. Another variable that can influence the color of vegetable lipid extracts is the presence of lipid oxidation reactions. These reactions commonly occur in lipid matrices, oils, and fats during storage, which can be measured by acidity and peroxide levels (presented previously in Table 4), as well as the presence of metallic ions, such as iron and copper (which can become active oxidizing agents) [33,35].

### 3.6. Thermogravimetric and Differential Analyses (TG-DTA)

**Figure 1:**
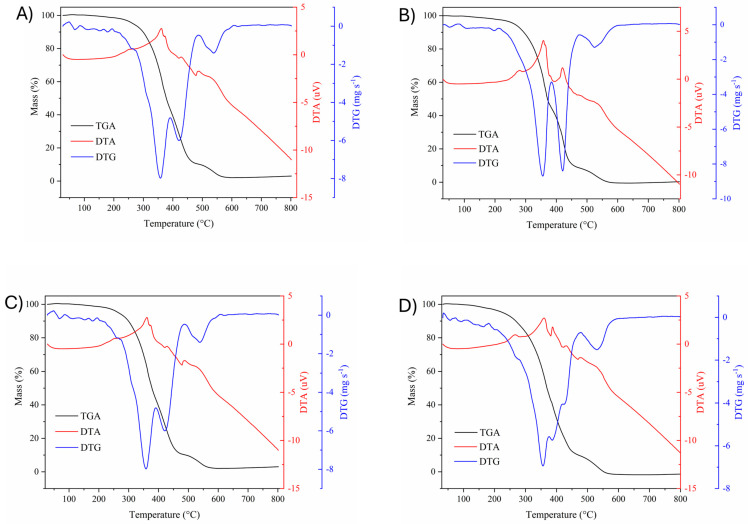
Thermogravimetric curves (TG/DTG and DTA) of the UAE lipid extracts from red (**A**), yellow (**B**), green (**C**), and white (**D**) peach palm fruits.

The thermogravimetric behavior of lipid extracts from peach palm varieties was studied and graphically represented in Figure 1A−D. The thermogravimetric (TG/DTG) and differential (DTA) behaviors of the lipid extracts showed differences in their respective mass losses along with a progressive increase in temperature.

All samples displayed a small mass decay around 100–250 °C, which is related to residual water content and volatile compounds [49]. The second mass loss presents a thermal degradation event occurring at around 350 °C for all samples, as can be seen by the mathematical derivative of the thermogravimetric curve (DTG). This decay is related to the initial decomposition temperature (T_onset_) that occurs at around 315 °C, while the peak temperature (T_peak_) occurs at around 355 °C, when the constituent acylglycerols and organic matter initiate its degradation. The samples from the red and yellow peach palm fruit varieties display a peak coupled to the T_peak_, like a shoulder, which can also be related to the degradation of organic compounds, and thus, the liberation of energy that accounts for the exothermal behavior of the samples [49]. The final mass loss occurred after 500 °C until 700 °C, after which around less than 20% of the carbonized matter remained.

It is well known that the degree of unsaturation, length, and branching of fatty acid chains influence the physico-chemical properties, as well as the thermal stability of lipids and oils, and can affect their industrial processability [50]. Hence, according to Li et al. [51], fatty acid systems and their esters with a higher unsaturation degree have lower oxidation stability because their unsaturation bonds need lower activation energy to initiate oxidation states, meaning that the fatty acid composition reliably reflects the system’s oxidation stability. In this context, it is possible to suggest that the lipid extracts from red and yellow peach palm fruits may be more susceptible to oxidation than the green and white samples, since the first two displayed unsaturated fatty acid contents above 75% and 68%, respectively, while the green and white lipid extracts displayed the highest proportions of saturated fatty acids, above 34% and 44%, respectively.

### 3.7. Infrared Spectroscopy

**Figure 2:**
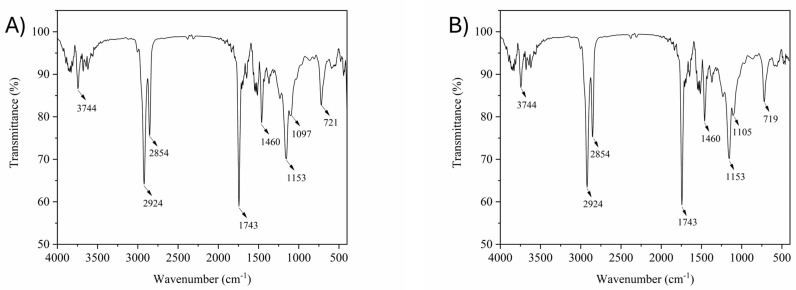

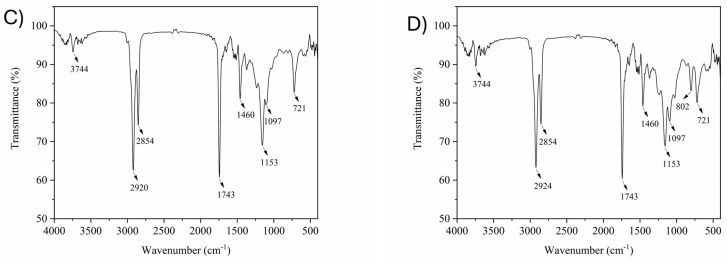
FTIR spectra of lipid extracts from red (**A**), yellow (**B**), green (**C**), and white (**D**) peach palm fruits.

Fourier-Transform Infrared Spectroscopy (FTIR) is an analytical tool useful for the identification of some chemical groups of substances by their spectral bands; it can also be used for the evaluation and prediction of the oxidation or adulteration status of vegetable oil matrices [52,53]. The band identification was conveyed according to the literature. The FTIR spectra of the lipid extracts from the four colors of peach palm fruits are presented in Figure 2.

We noted the presence of the same spectral frequency bands for all peach palm lipid extracts. The FTIR spectra of the peach palm lipid extracts shown in Figure 2A−D, present typical absorption bands of triglycerides and FAs. The bands near 2920–2924 cm^−1^ were attributed to stretching vibration of methylene (−CH_2_), with similar intensities for all samples, as functional groups that were characteristic of long-chain fatty acids, such as unsaturated [52]. Linolenic acyl groups (C18:2) were reported to exhibit a high frequency for this band region [54]. Vibration bands in the range of 1650–1750 cm^−1^ were detected in all samples, which could be related to the stretching vibration of triacylglycerol ester linkages (–C=O) [52,55]. The bands in the absorption ranges from 1153 cm^−1^ to 1097 cm^−1^, common to all samples evaluated, can be linked to –C−O stretching and are commonly perceived as the fingerprint region of samples [54,56].

The high-intensity band at 1153 cm^−1^ can be observed in all samples, which can be attributed to a high amount of oleic acyl groups in the samples [54]. It is worth saying that the band at 965 cm^−1^, related to the out-of-plane bending vibration of the trans-disubstituted olefins, was not detected in any peach palm samples, which confirmed only the presence of cis conformations of the CH=CH bonds in the unsaturated fatty acids in the samples [57]. The smallest bands at 802 cm^−1^ to 721 cm^−1^ may also be related to the bending vibrations of C–H (out of plane vibration of cis-disubstituted olefin) and saturated carbon–carbon bonds [52,56]. Moreover, the FTIR results agree with the FA profiles determined in the present work.

## 4. Conclusions

The present study showed that the lipid extracts obtained by ethanolic-based ultrasound-assisted extraction from the four different colors of peach palm fruit—red, yellow, green, and white—had lower levels of acidity and peroxides, which agrees with the standards recommendations for edible oils. The red peach palm lipid extract presented the highest content of unsaturated fatty acids, mostly oleic acid, while the white lipid extract presented the highest content of saturated fatty acid among the samples. This diverse content of fatty acids influenced the thrombogenicity and atherogenicity indices, as well as the hypocholesterolemic/hypercholesterolemic ratio, which were the greatest in the red peach palm lipid extract. Regarding the total carotenoids, once again, the red peach palm lipid extract presented the highest content. The infrared spectroscopic profiles of all four samples displayed low-intensity frequency bands related to their oxidation states. All peach palm extracts had good thermal stability, also agreeing with the fatty acid composition. The use of a green extraction method and the characterization of the peach palm lipid extracts were relevant to adding value to new sources of vegetable lipid extracts with diverse industrial applications and the development of sustainable and functional products. Despite the low extraction yield, ethanolic ultrasound-assisted extraction was effective in obtaining peach palm lipids with good physicochemical, nutritional, and functional qualities, as well as with relevant thermal stability, to be used as a novel source and sustainable source of good-quality lipids for future incorporation into food products. Moreover, additional studies are needed to study optimum extraction conditions, as well as the application of blended methods to obtain novel products.

## Figures and Tables

**Table 1:** Extraction yield of lipids from the pulp of different peach palm fruit colors.

	Lipid Extracts from Four Colours of Peach Palm Fruits			
Yield of Lipids (%)	Red	Yellow	Green	White
	8.85 ± 0.95	7.35 ± 0.83	3.35 ± 0.75	2.15 ± 0.17

The results are expressed as mean ± standard deviation (*n* = 3).

**Table 2:** Profile of fatty acids from peach palm pulp lipid extract obtained by using ultrasound-assisted extraction.

	Lipid Extracts from Four Colours of Peach Palm Fruits			
Fatty Acid Profile (%)	Red	Yellow	Green	White
Saturated Fatty Acids—SFAs				
Lauric acid (12:0)	0.01 ± 0.00 ^a^	0.02 ± 0.00 ^b^	0.01 ± 0.00 ^a^	0.01 ± 0.00 ^a^
Myristic acid (14:0)	0.08 ± 0.00 ^a^	0.15 ± 0.00 ^b^	0.08 ± 0.00 ^a^	0.10 ± 0.00 ^b^
Palmitic acid (16:0)	23.77 ± 0.15 ^a^	28.96 ± 0.23 ^b^	33.86 ± 0.34 ^c^	42.62 ± 0.43 ^d^
Stearic acid (18:0)	Nd	0.70 ± 0.02 ^a^	Nd	1.87 ± 0.23 ^b^
Arachidic acid (20:0)	0.14 ± 0.02 ^a^	0.10 ± 0.00 ^a^	0.12 ± 0.03 ^a^	0.19 ± 0.00 ^a^
Monounsaturated Fatty Acids—MUFAs				
Palmitoleic acid (16:1)	9.89 ± 0.34 ^a^	13.23 ± 0.12 ^b^	3.98 ± 0.91 ^c^	4.99 ± 0.03 ^c^
Oleic acid (18:1n-9)	60.20 ± 0.50 ^a^	44.85 ± 0.41 ^b^	57.62 ± 0.14 ^c^	40.73 ± 0.54 ^d^
Polyunsaturated Fatty Acids—PUFAs				
Linoleic acid (18:2n-6)	4.04 ± 0.61 ^a^	8.05 ± 0.91 ^b^	2.03 ± 0.14 ^c^	6.95 ± 0.17 ^d^
Alpha-linolenic acid (18:3n-3)	1.48 ± 0.24 ^a^	2.50 ± 0.07 ^b^	0.54 ± 0.23 ^c^	2.14 ± 0.34 ^b^
∑ SFAs	24.012	29.946	34.086	44.808
∑ MUFAs	70.097	58.086	61.60	45.730
∑ PUFAs	5.521	10.556	2.578	9.097
PUFAs/SFAs	0.230	0.352	0.075	0.203

Nd = not determined. Data are expressed as mean ± standard deviation. Different lower-case letters in the same row indicate significant statistical differences by Tukey’s test (*p* < 0.05).

**Table 3:** Nutritional-quality indices and total carotenoids of peach palm lipid extract obtained by ultrasound-assisted extraction.

Lipid Extracts from Four Colours of Peach Palm Fruits				
Nutritional-Quality Indices	Red	Yellow	Green	White
AI	0.35	0.47	0.53	0.78
TI	0.52	0.72	0.92	1.34
H/H	2.75	1.90	1.77	1.16
Total carotenoids (μg of β-caroten 100 g^−1^ of lipid extract)	748 ± 12.75	247 ± 8.63	207 ± 7.27	32 ± 3.33

Data are expressed as mean ± standard deviation. AI = atherogenicity index, TI = thrombogenicity index, and H/H = hypocholesterolemic/hypercholesterolemic ratio.

**Table 4:** Physicochemical parameters of UAE lipid extracts from different colors of peach palm fruits.

Lipid Extracts from Four Colors of Peach Palm Fruits				
Parameters	Red	Yellow	Green	White
Acidity (mg KOH g^−1^)	0.85 ± 0.55 ^a^	0.78 ± 0.15 ^a^	0.82 ± 0.25 ^a^	1.28 ± 0.35 ^b^
Peroxide (mEq kg^−1^)	1.75 ± 0.35 ^a^	1.87 ± 0.23 ^a^	1.85 ± 0.65 ^a^	2.59 ± 040 ^b^

Data are expressed as mean ± standard deviation. Different lower-case letters in the same column indicate significant statistical differences by Tukey’s test (*p* < 0.05).

**Table 5:** Instrumental colorimetric results for the lipid extracts from four colors of peach palm fruits using the CIELAB system.

Lipid Extracts from Four Colours of Peach Palm Fruits				
Parameters	Red	Yellow	Green	White
L*	27.1 ^b^	28.36 ^b^	29 ^ab^	38.8 ^a^
a*	11.4 ^a^	6.3 ^b^	5.4 ^c^	2.3 ^d^
b*	9.7 ^b^	11.43 ^a^	10.16 ^ab^	4.2 ^c^
ΔE	71.71	70.07	69.20	58.78
Croma	14.97	13.05	11.50	4.79
Hue angle (h_ab_)	40.39	61.14	62.01	61.29
Whiteness index (WI)	25.57	27.18	28.08	38.61

L* = luminosity (black = 0 and white = 100), a* = chromaticity (+a = red and −a = green), b* = chromaticity (+b = yellow and −b = blue), and ΔE = geometric distance between the color of the sample to a reference. Different lower-case letters in the same line indicate significant statistical differences by Tukey’s test (*p* < 0.05).

## Data Availability

The original contributions presented in the study are included in the article, further inquiries can be directed to the corresponding author.
